# YKL-40 promotes chemokine expression following drug-induced liver injury via TF-PAR1 pathway in mice

**DOI:** 10.3389/fphar.2023.1205062

**Published:** 2023-08-24

**Authors:** Zhan Jing-Lun, Chai Shuang, Zhao Li-Mei, Liu Xiao-Dong

**Affiliations:** ^1^ Department of Pharmacy, Shengjing Hospital of China Medical University, Shenyan, China; ^2^ Department of the Second Clinical Pharmacy, School of Pharmacy, China Medical University, Shenyang, China

**Keywords:** YKL-40, TF-PAR1 pathway, inflammation, liver injury, CCL2, IP-10

## Abstract

**Background:** The inflammatory factor YKL-40 is associated with various inflammatory diseases and is key to remodeling inflammatory cells and tissues. YKL-40 (Chi3l1) promotes the activation of tissue factor (TF), leading to intrahepatic vascular coagulation (IAOC) and liver injury. TF is a key promoter of the exogenous coagulation cascade and is also involved in several signaling involving cell proliferation, apoptosis, charring, migration and inflammatory diseases pathways. However, the effect of YKL-40-induced TF-PAR1 pathway on the expression of downstream chemokines remains unknown.

**Methods:** We established a liver injury model using Concanavalin A (ConA) in C57 BL/6 mice. By adopting various experimental techniques, the effect of YKL-40 induced TF-PAR1 pathway on the expression of downstream chemokine ligand 2 (CCL2) and IP-10 was verified.

**Results:** We found that overexpression of YKL-40 increased the expression of TF, protease-activated receptor 1 (PAR1), CCL2 and IP-10 in mice and exacerbated the severity of liver injury. However, blocking the expression of TF significantly reversed the extent of liver injury.

**Conclusion:** We found that YKL-40 promotes the expression of downstream chemokines ligand 2 (CCL2) and IP-10 by activating the TF-PAR1 pathway, leading to increased recruitment of inflammatory cells and exacerbating the progression of liver injury. This provides a new approach for the clinical treatment of drug-induced liver injury.

## 1 Introduction

Drug-induced liver injury (DILI) is the primary cause of acute liver failure and poor outcomes of transplantation. The annual incidence rate of DILI has increased recently, outpacing fatty liver disease and viral hepatitis (Katarey and Verma, 2016). Several studies have revealed that intrahepatic vascular coagulation (IAOC) is a major cause of the onset and progression of liver fibrosis, cirrhosis, and liver cancer (Ganey et al., 2007; Sullivan et al., 2013; Miyakawa et al., 2015), and the inflammatory factor YKL-40 (Chi3l1) is associated with various inflammatory diseases. YKL-40 levels are increased to varying degrees in infectious diseases, such as respiratory, kidney, liver, cardiovascular, and skin inflammatory diseases (Montgomery et al., 2017; Ma et al., 2018; Shan et al., 2018; Arain et al., 2020; Ebrahim et al., 2020), and it has diagnostic as well as prognostic value as an inflammatory marker.

YKL-40 is a member of the 18-glycosyl hydrolase family without chitinase activity. It is expressed in several cells, including megaphage/monocytes, neutrophils, fibroblasts, vascular smooth muscle cells, and chondrocytes (Johansen, 2006; Lee et al., 2011). Overexpression of YKL-40 is linked to the pathogenesis and prognosis of several inflammatory diseases. YKL-40 affects angiogenic activity, inflammatory microenvironment, and epithelial-mesenchymal transformation, by promoting the release of inflammatory factors, resulting in the activation of corresponding signal transduction pathways (Montgomery et al., 2017; Ma et al., 2018; Shan et al., 2018; Arain et al., 2020; Ebrahim et al., 2020). Studies in murine model of concanavalin (ConA)-induced liver injury have revealed that YKL-40 (Chi3l1) promotes the activation of tissue factor (TF), thereby inducing IAOC and eventually leading to liver injury (Shan et al., 2018). However, the mechanism underlying TF-mediated activation of IAOC that exacerbates liver disease is not fully understood.

Coagulation dysfunction caused by liver injury is frequently associated with upregulated TF expression (Hisada et al., 2016; Henderson et al., 2021). The dysregulated coagulation cascade has been the main pathophysiological manifestation of TF activation. Furthermore, TF activity impacts procoagulant events and inflammatory non-coagulation signaling pathways transmitted through specific receptors, such as PARs (protease-activated receptors) pathway. During an inflammatory disease, inflammatory factors induce TF release into the blood, where it causes coagulation cascade reaction, forming a vicious circle of “coagulation-inflammation network,” exacerbating the disease (Witkowski et al., 2016). The histological grade of the illness and the TF levels are strongly correlated. The elevated TF expression significantly impacts patient prognosis and survival rate. Mounting evidence suggests that the TF-PARs pathway can promote the progression of inflammatory diseases, cancer, and other diseases. However, the specific mechanism underlying TF-PARs pathway and its target recognition remain largely unknown. Protein kinase 1 (PAR1), PAR2, PAR3, and PAR4 are members of the PARs family and TF affects cell proliferation, apoptosis, scorching, migration, and the onset and development of inflammatory diseases through the TF-PAR1 pathway (Reinhardt et al., 2012; Feng et al., 2020).

Chemokines play an important role in the pathophysiology of autoimmune hepatitis, development of liver inflammation, and subsequent wound healing response. Chemokines regulate the movement and activity of liver cells including Kupffer cells and hepatic stellate cells, endothelial cells, and circulating immune cells and guide the movement and positioning of inflammatory and immune cells in the liver (Zimmermann and Tacke, 2011). Studies have revealed that hepatitis is directly related to abnormal chemokine production (Fahey et al., 2014). Chemokine ligand 2 (CCL2) and chemokine IP-10 (also known as CXCL10) are inflammatory chemokines that play a key role in recruiting neutrophils and promoting the secretion of various other cytokines, thereby aggravating disease occurrence and progression (Mascia et al., 2017; Tacke, 2017). Notably, CCL2 is one of the chemokines involved in liver fibrosis. Chronic chemokine production may result in a continuous assemblage of inflammatory cells in the liver, causing persistent inflammation and liver damage (Kang and Shin, 2011).

Here, we used a mouse model of ConA-induced liver injury to investigate how YKL-40 promotes TF activation and influences the occurrence and progression of DILI. We show that YKL-40 promotes the expression of CCL2 and IP-10 by inducing the TF-PAR1 pathway in the liver, leading to an increase in inflammatory cell recruitment, thus sparking the onset and progression of liver injury. Furthermore, TF and PAR1 expression levels positively correlated with YKL-40 expression levels following the ConA induction in our study. Thus, YKL-40 is the potential key upstream effector of the TF-PAR1 pathway and promotes the progression of liver injury.

## 2 Materials and methods

### 2.1 Animal experiments

Male C57/BL6 mice (8–12 weeks old) were used in the study. Mice were obtained from Beijing Huafukang Biotechnology Co., Ltd. (Beijing, China) and housed in a temperature-and light-controlled laboratory with *ad libitum* access to food and water. Animal experiments were carried out in accordance with the national standard of the People’s Republic of China, Guidelines for Ethical Review of Laboratory Animal Welfare (GB/T35892-2018) and the guidelines of the Animal Protection and Use Committee of China Medical University. Briefly, C57/BL6 mice were divided into 4 experimental groups. To establish a mouse model of DILI, 15 mg/kg ConA (Solarbio, C8110) was injected into the tail vein of mice. In the experimental groups, mice were immediately injected with recombinant Chi3l1 (500 ng, Sino Biological, 50929-M08H), anti-Chi3l1 antibody (500 ng, Sino Biological, 50929-RP01), or anti-TF antibody (1/1,000, 500 μg, Bioss, bs-4690R) after receiving ConA (Shan et al., 2018). Finally, mice were anesthetized with phenobarbital, their livers were collected, and subjected to further analysis.

### 2.2 ELISA

The experiment was performed using a mouse YKL-40 ELISA kit (Shanghai Shuangying Biotechnology Co., Ltd., SY-M06, 294, China) per manufacturer’s protocol.

### 2.3 Real-time PCR

RIPA lysis buffer (TransGen Biotech, AU341, China) was used to extract RNA from real-time PCR. Quantitative real-time PCR was used to analyze gene expression using QuantiStudio 1. The following primers were used.

**Table:** 

Gene	Primer sequences
TF	Forward:AACCCACCAACTATACCTACACT
	Reverse:GTCTGTGAGGTCGCACTCG
PAR1	Forward:GGCGCTTGCTGATCGTC
	Rreverse:CGTAGCATCTGTCCTCTCTGA
CCL2/MCP-1	Forward:TTAAAAACCTGGACTGGAACCAA
	Reverse:GCATTAGCTTCAGATTTACGGGT
CXCL10/IP-10	Forward:TGAATCCGGAATCTAAGACCATCAA
	Reverse:AGGACTAGCCATCCACTGGGTAAAG
GAPDH	Forward:GCTACACTGAGGACCAGGTTGTC
	Reverse:AGCCGTATTCATTGTCATACCAGG

### 2.4 Western blot analysis

We weighed liver tissues and extracted the protein according to the standard protocol. Protein samples were resolved by performing SDS-polyacrylamide gel electrophoresis and transferred to a membrane. The membrane was then blocked with a rapid blocking solution and incubated with the specific primary antibodies against TF (Bioss, bs-4690R, 1/1,000), PAR1 (Solarbio, K009690P, 1/1,500), CCL2/MCP-1 (ProteinTech, 25542-1-AP, 1/2,000), and CXCL10/IP-10 (ProteinTech, 10937-1-AP, 1/500). GAPDH (ProteinTech, 10494-1-AP, 1/6000) was used as the control. This was followed by incubation with the secondary antibody (goat anti-rabbit IgG (H+L); ProteinTech, SA00001-2, 1:6,000). Finally, immunoreactive bands were visualized using a chemiluminescent solution and chemiluminescence imaging system. The immunoreactive bands were quantified using ImageJ software.

### 2.5 Immunohistochemical (IHC) staining

Liver tissue samples were fixed in 4% paraformaldehyde, embedded in paraffin (KEDEE,KD-BM,China), and sectioned (HistoCore AUTOCUT, China). Slice thickness is 5 um. The primary antibodies against TF (ProteinTech, 17435-1-AP, 1:3,000), PAR1 (Solarbio, K009690P, 1/1500), CCL2/MCP-1 (ProteinTech, 25542-1-AP, 1/2000), and CXCL10/IP-10 (ProteinTech, 10937-1-AP, 1/500) were used and signals were quantified.

### 2.6 Hematoxylin & eosin (H&E) staining

The embedded liver tissue sections were dewaxed and stained with H&E staining kit (Solarbio, G1120, China). The slices were stained with eosin for 1 min and after 10 min of hematoxylin staining, they were subjected to 1 min of differentiation using the differentiation solution. The slices were then dried in a series of graded alcohol and treated with xylene twice for 1 min each. The slides were sealed with neutral gum and examined under a microscope.

### 2.7 Statistical analysis

The data is presented as the mean ± SEM. GraphPad Prism (GraphPad Software) was used for statistical analysis. The unpaired Student’s t-test was used to evaluate the differences between the two groups. A one-way analysis of variance was used to examine the results from several groups. Results with *p* < 0.05 were considered statistically significant.

## 3 Results

### 3.1 YKL-40 exacerbates the progression of liver injury

**FIGURE 1:**
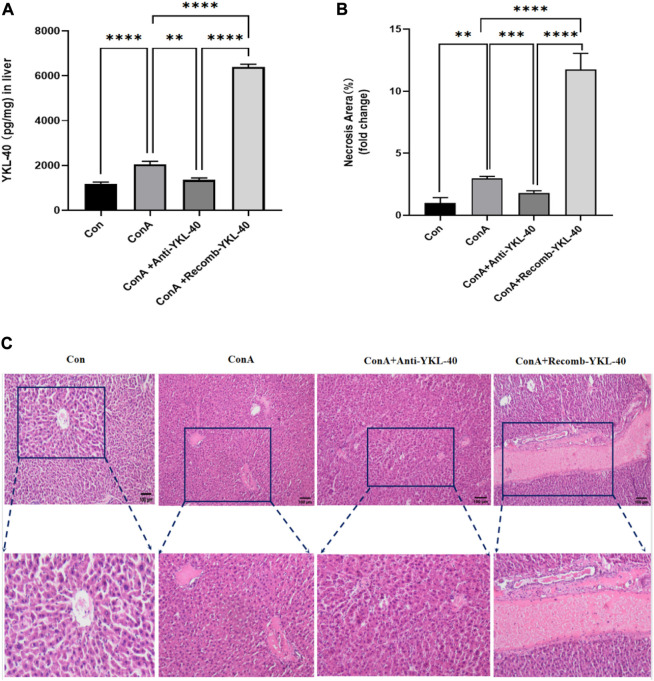
YKL-40 exacerbates the progression of liver injury.**(A)** The expression of YKL40 in liver tissue; **(B)** Hematoxylin and Eosin staining (magnification×100) showing YKL-40-mediated induction of TF-PAR1 pathway and aggravation of drug-induced liver injury. **(C)** Necrotic areas as quantified by ImageJ software. Data are expressed as mean ± SEM (n = 6). ^*^ *p* < 0.05, ^**^ *p* < 0.01, ^***^ *p* < 0.001, ^****^ *p* < 0.0001, ns means no statistical difference.

Firstly, we induced high and low expression of YKL40 to elucidate the relationship between YKL-40 expression and the course of drug-related liver injury, and further clarify the degree of liver injury in each experimental group. We use ELISA and HE staining techniques. The ELISA results showed that after ConA induction, TF and PAR1 levels were positively correlated with YKL-40 levels (Figure 1A). Then, we examined the tissue damage at the histological level. It should be noted that in Con group, complete Lobules of liver structure was shown in HE staining. On the contrary, in ConA group, the structure of Lobules of liver was destroyed and disordered, and Vasodilation was observed, there was obvious red blood cell sludge in hepatic sinuses, liver edema, especially in the portal vein area, and a large number of lymphocytes. More importantly, more serious liver injury was observed in the YKL-40 overexpression group, which was manifested as structural destruction of the Lobules of liver, large and deeply stained liver nuclei, dual nuclei, diffuse cytoplasm, obvious inflammation in the portal vein area, liver edema, and severe congestion of the hepatic sinuses around the central vein with patchy or lamellar necrosis. On the contrary, when YKL-40 is low expressed, Lobules of liver structure damage, hepatic cord disorder and hepatic tissue congestion are relatively less (Figures 1B,C). In summary, it can be seen that the degree of liver injury often increases with the increase of YKL-40 expression.

### 3.2 YKL-40 induces TF-PAR1 pathway and promotes CCL2 and IP-10 expression

**FIGURE 2:**
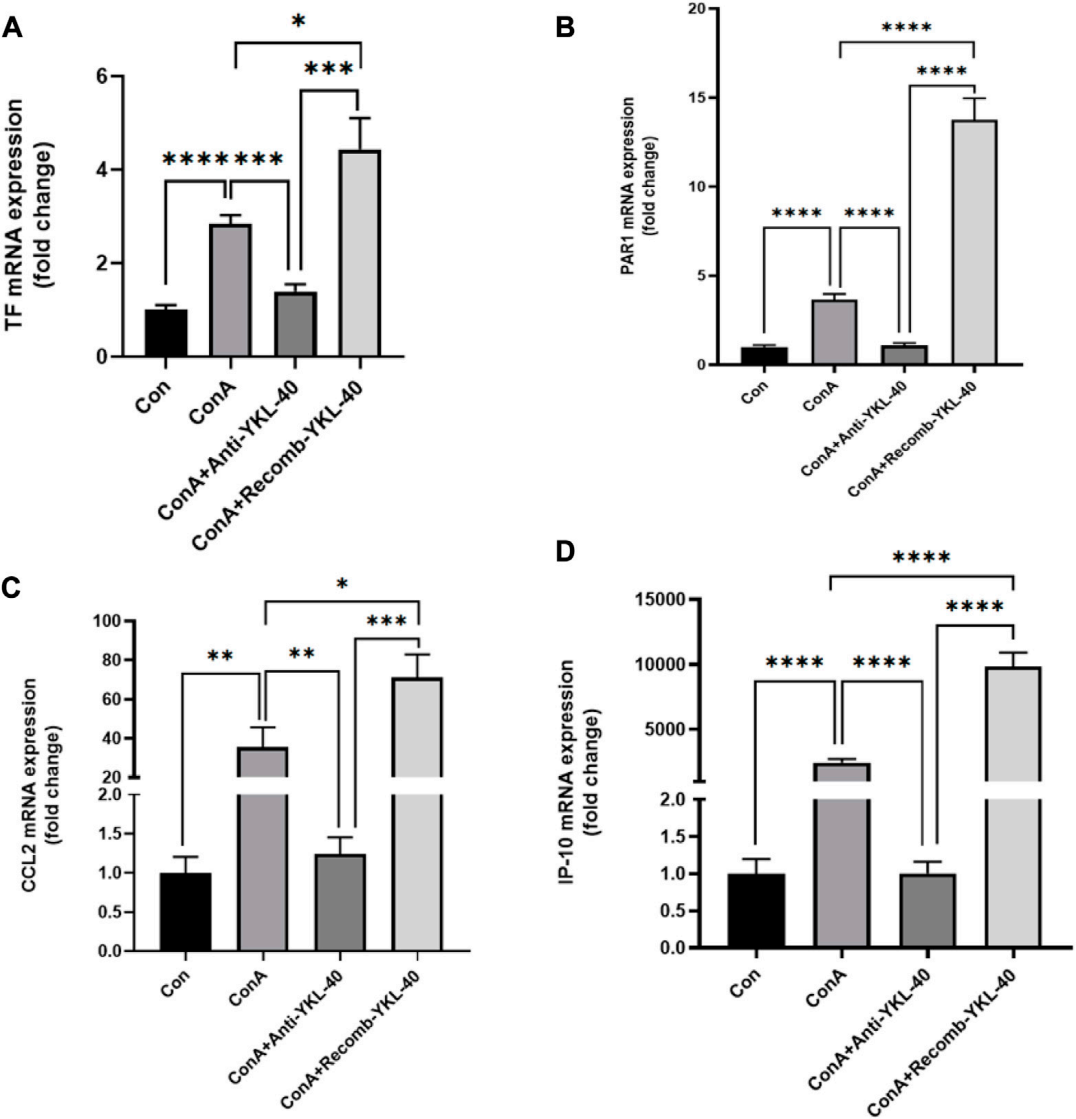
YKL-40 induces TF-PAR1 pathway, promoting the expression of chemokines CCL2 and IP10. **(A)** TF mRNA expression in mouse liver; **(B)** PAR1 mRNA expression in mouse liver; **(C)** CCL2 mRNA expression in mouse liver; **(D)** IP-10 mRNA expression in mouse liver. *p* values were determined using one-way ANOVA or an unpaired *t*-test. Data are expressed as mean ± SEM (n = 6). ^*^ *p* < 0.05, ^**^ *p* < 0.01, ^***^ *p* < 0.001, ^****^ *p* < 0.0001, ns means no statistical difference.

**FIGURE 3:**
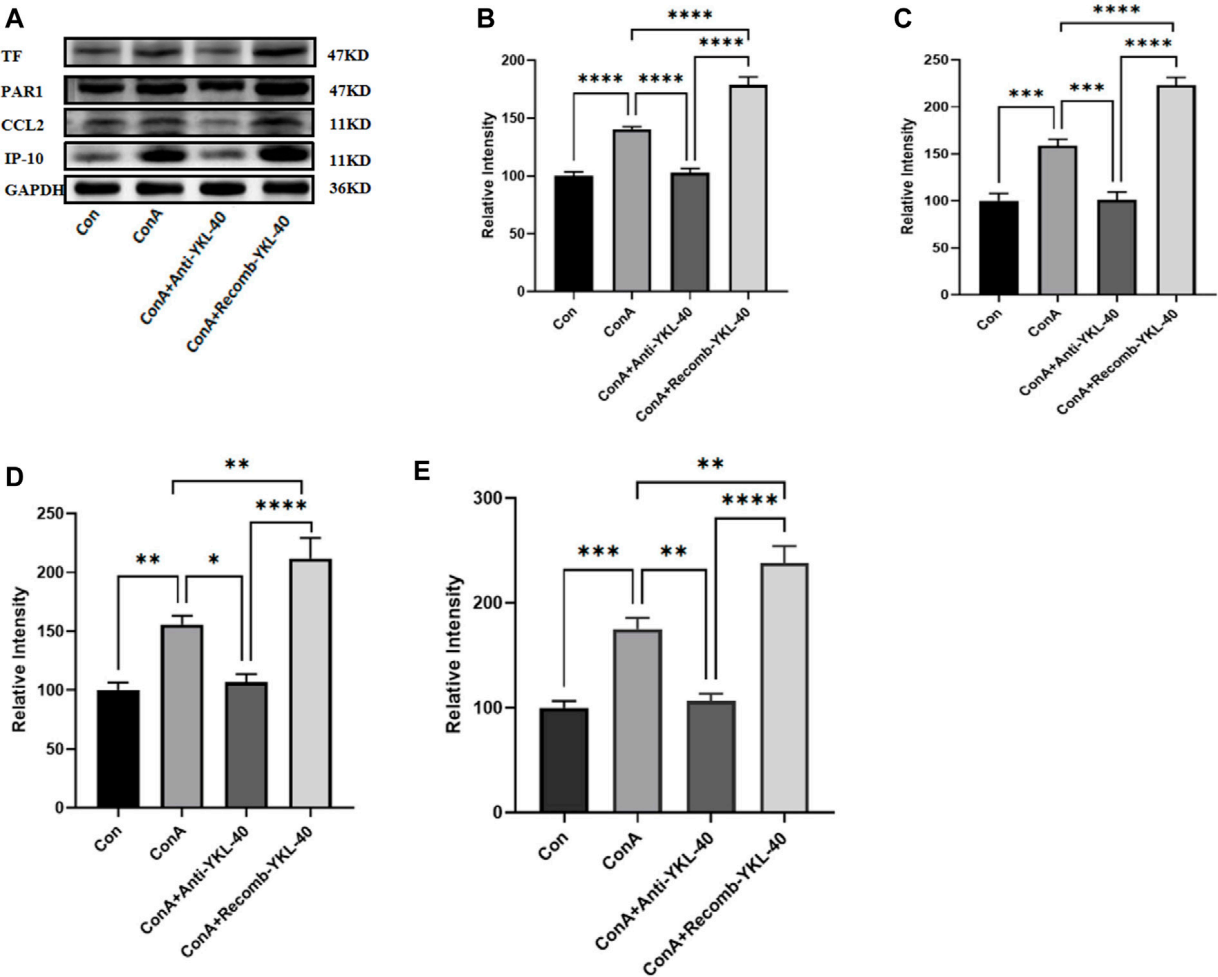
YKL-40 induces TF-PAR1 pathway and affects liver pathogenesis. **(A)** The levels of TF-PAR1 pathway proteins induced by YKL-40 in the liver and CCL2 and IP-10 were measured; **(B)** TF protein expression in mouse liver; **(C)** PAR1 protein expression in mouse liver; **(D)** CCL2 protein expression in mouse liver; **(E)** IP-10 protein expression in mouse liver. *p* values were determined using one-way ANOVA or an unpaired t-test. Data are expressed as mean ± SEM (n = 6). *p < 0.05, **p < 0.01, ***p < 0.001, ****p < 0.0001, ns means no statistical difference.

PAR1 is the main receptor for thrombin, closely related to TF expression, and involved in liver inflammation and fibrosis (Gutiérrez-Rodríguez and Herranz, 2015; Flaumenhaft and De Ceunynck, 2017). Thrombin activates PAR1 in endothelial cells, upregulates adhesion molecules on the surface of endothelial cells, and promotes the production of cytokines and chemokines, thus activating neutrophils and Monocyte. Our real-time PCR data shows that an increase in TF, PAR1, CCL2, and IP-10 levels is associated with an increase in YKL-40 expression level (Figures 2A–D). The results of immunoblotting are consistent with these findings (Figure 3).

### 3.3 YKL-40-mediated induction of TF-PAR1 pathway aggravates DILI progression

**FIGURE 4:**
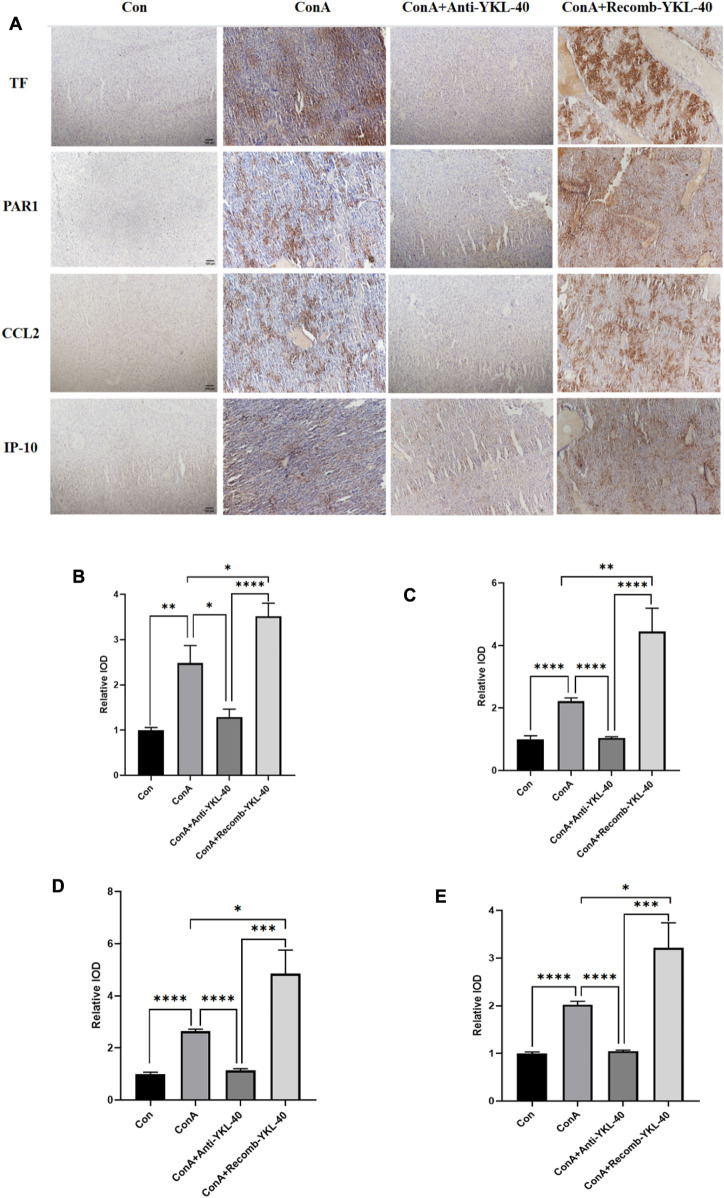
YKL-40-mediated induction of TF-PAR1 pathway aggravates DILI progression. **(A)** Immunohistochemistry (IHC) (magnification×100) was performed to determine whether YKL-40 induced TF-PAR1 pathway and aggravated drug-induced liver injury; **(B)** Immunohistochemical statistics of TF; **(C)** Immunohistochemical statistics of PAR1; **(D)** Immunohistochemical statistics of CCL2; **(E)** Immunohistochemical statistics of IP-10. *p* values were determined using one-way ANOVA or an unpaired *t*-test. Data are expressed as mean ± SEM (n = 6). ^*^ *p* < 0.05, ^**^ *p* < 0.01, ^***^ *p* < 0.001, ^****^ *p* < 0.0001, ns means no statistical difference.

CCL2 is involved in the pathogenesis of many liver diseases, such as hepatitis, fibrosis, and cancer. Research has found that the PAR1 signaling pathway affects the expression of CCL2, which in turn promotes the recruitment of neutrophils in inflammatory diseases. In severe acute hepatitis mice that experience necrotizing inflammation and acute Liver failure, IP-10 may be important for lymphocyte recruitment (Reiberger et al., 2008). Here, we conducted histological examination to investigate the role of YKL-40 mediated TF-PAR1 pathway in the progression of DILI. Immunohistochemical and other experimental results showed that the expression of TF, PAR1, CCL2, and IP-10 in the liver of drug-induced liver injury mice in the ConA group was higher than that in the untreated group and the YKL-40 low expression group. However, when YKL-40 was overexpressed, the expression was significantly higher than the other three groups. Therefore, YKL-40 induces the TF-PAR1 pathway, significantly increasing the expression of downstream chemokines CCL2 and IP-10 (Figure 4).

### 3.4 YKL-40-mediated TF-PAR1 pathway can effectively alleviate the progression of DILI after being blocked

**FIGURE 5:**
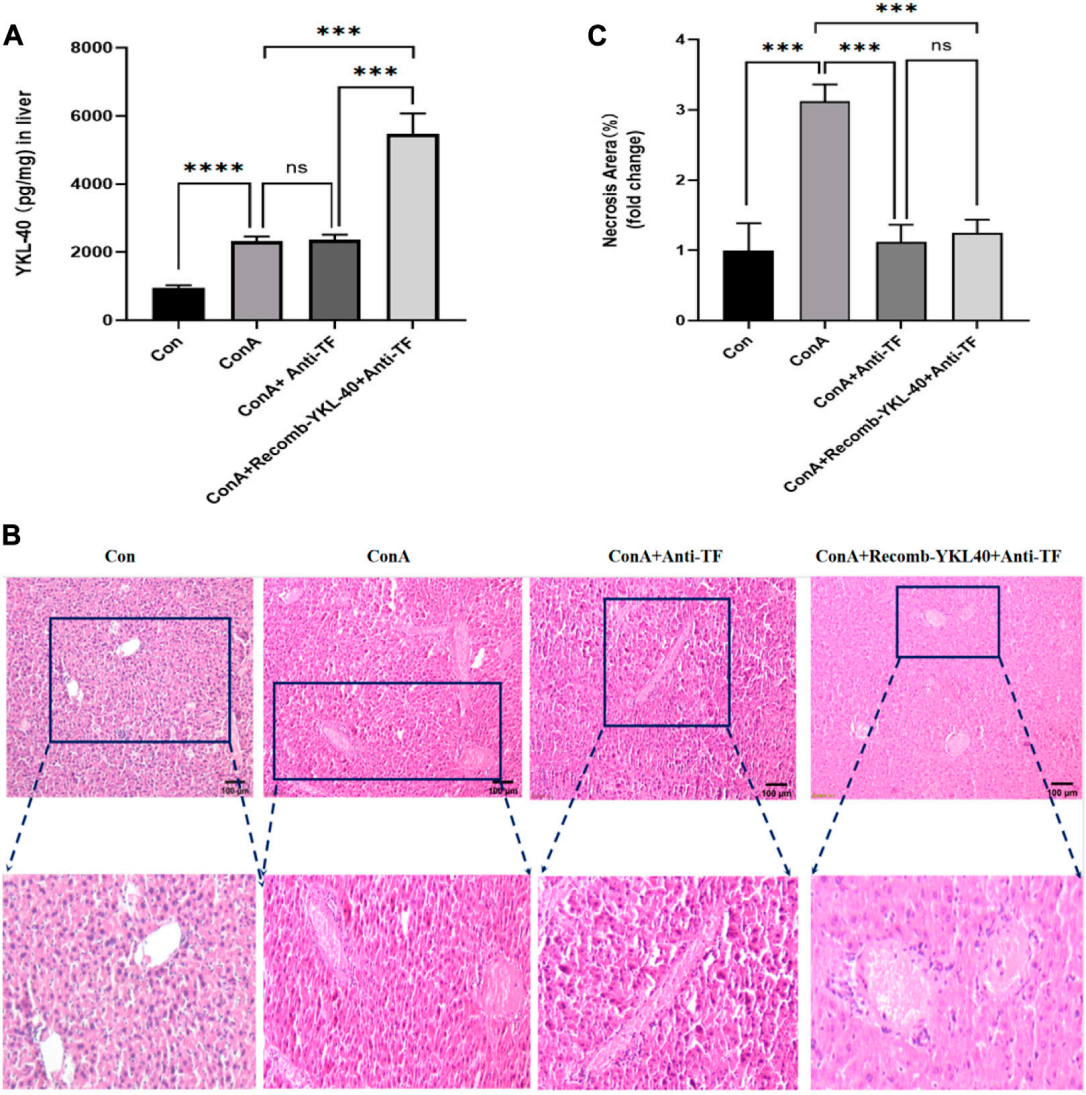
YKL-40-mediated TF-PAR1 pathway can effectively alleviate the progression of DILI after being blocked. **(A)** After the endogenous TF protein expression was blocked YKL-40 expression in liver tissue; **(B)** Blocking TF suppressed YKL-40-induced TF-PAR1 pathway and mitigated drug-induced liver injury (magnification×100). **(C)** Necrotic areas as quantified by ImageJ software. Data are expressed as mean ± SEM (n = 6). ^*^ *p* < 0.05, ^**^ *p* < 0.01, ^***^ *p* < 0.001, ^****^ *p* < 0.0001, ns means no statistical difference.

To investigate the effect of the TF-PAR1 pathway on the expression of CCL2 and IP-10, we used anti TF antibodies to inhibit endogenous TF proteins. Use ELISA to detect the expression of YKL-40 in each group. Research has found that when the TF-PAR1 pathway is blocked, there is no statistically significant difference in the expression of YKL-40 compared to the drug-induced liver injury group caused by ConA. It is worth noting that even in the medium, the expression of YKL-40 was not affected and remained in a high expression state (*p* < 0.001), indicating that YKL-40 acts as an upstream of the TF-PAR1 pathway and is not affected by blocking the TF-PAR1 pathway (Figure 5A). Then, we used H&E stain to explore and study the histological examination method. The results showed that the structure of Lobules of liver in Con group was intact; In ConA group, the structure of Lobules of liver was damaged, hepatic cord was disordered, hepatocytes were edematous, and there was obvious red cell sludge and inflammatory cell infiltration in hepatic sinuses; In the liver tissue of ConA+Anti TF and ConA+RecombYKL-40+Anti TF groups, the structure of Lobules of liver was slightly damaged, the arrangement of hepatic cords was slightly disordered, a small amount of congestion was found in the liver tissue, and the hepatic sinuses around the central vein were congested (Figures 5B,C). In summary, blocking TF expression can effectively alleviate the progression of DILI.

### 3.5 YKL-40-mediated induction of TF-PAR1 pathway affects the course of DILI by regulating TF expression

**FIGURE 6:**
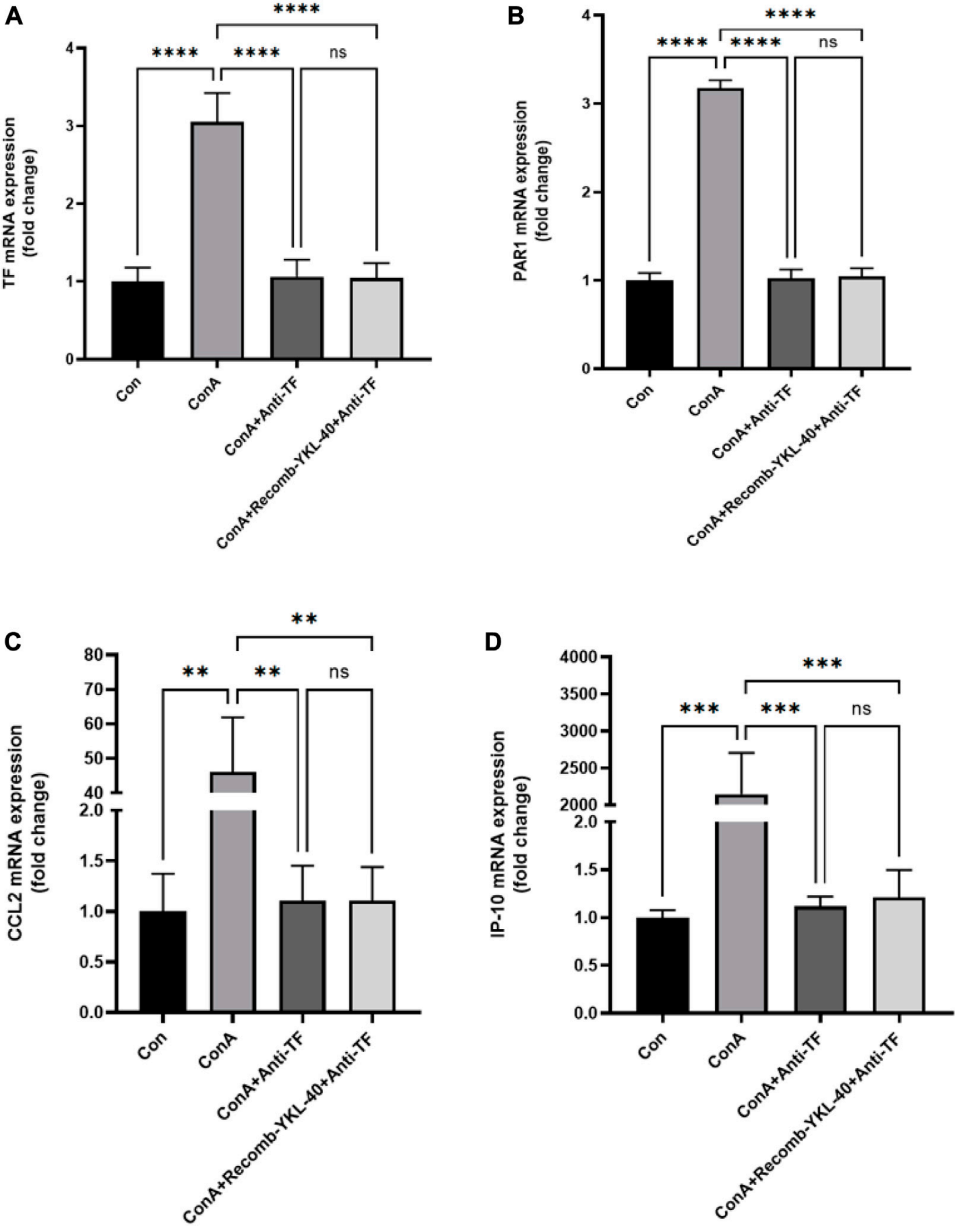
YKL-40-induced TF-PAR1 pathway influences the course of DILI by regulating TF expression. **(A)** After blocking the endogenous TF protein expression, TF mRNA expression was measured in mouse liver; **(B)** After blocking the endogenous TF protein expression, PAR1 mRNA was measured in mouse liver; **(C)** After blocking the endogenous TF protein expression, CCL2 expression was measured in mouse liver; **(D)** After blocking the endogenous TF protein expression, IP-10 expression was measured in mouse liver. *p* values were determined using one-way ANOVA or an unpaired *t*-test. Data are expressed as mean ± SEM (n = 6).^*^ *p* < 0.05, ^**^ *p* < 0.01, ^***^ *p* < 0.001, ^****^ *p* < 0.0001, ns means no statistical difference.

**FIGURE 7:**
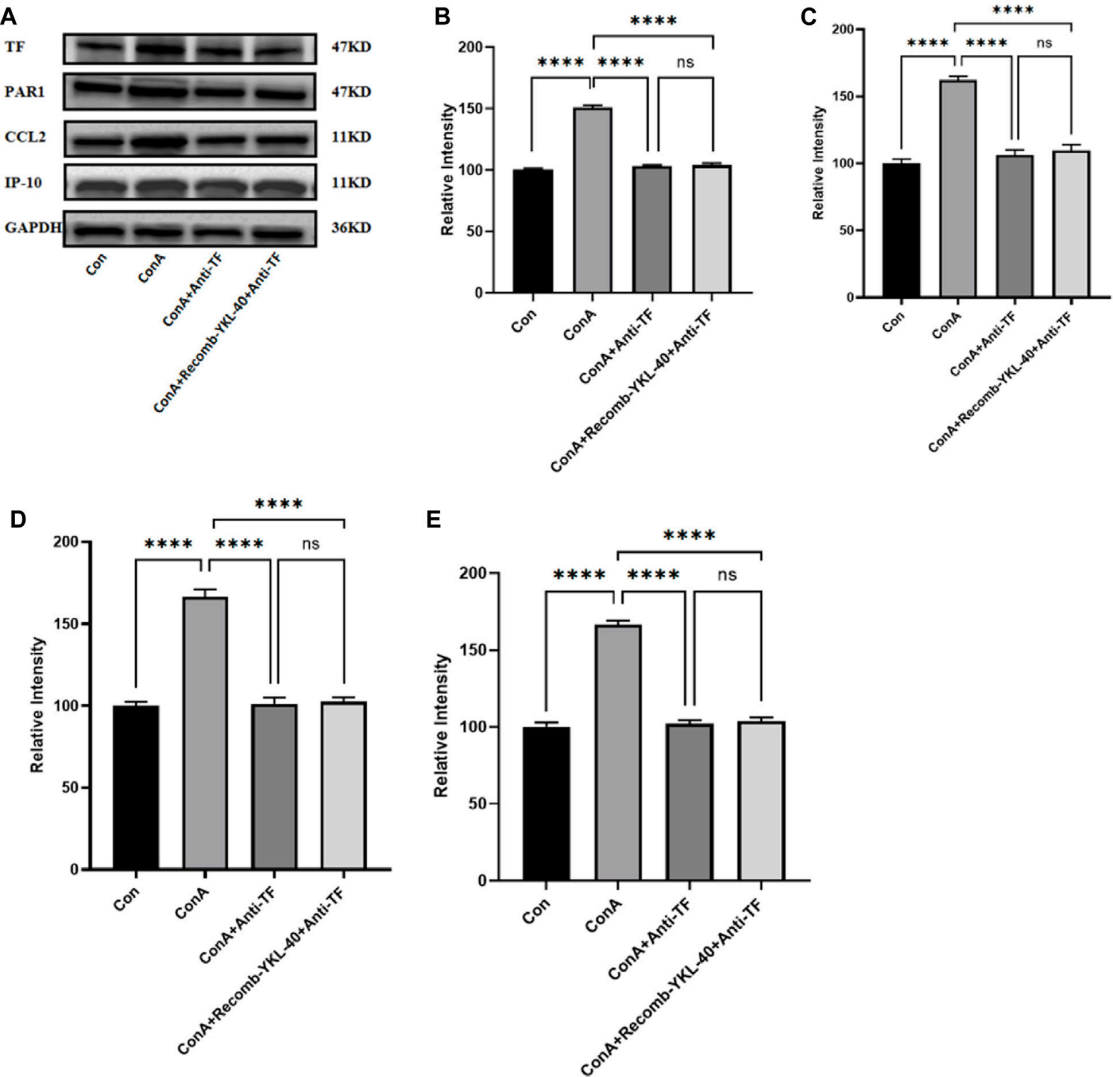
Effect of blocking TF-PAR1 pathway on liver pathogenesis. After the endogenous TF protein expression was blocked, **(A)** the expression of TF-PAR1 pathway proteins in the liver following YKL-40 induction and the levels of downstream chemokines CCL2 and IP-10 were determined; **(B)** TF protein expression in mouse liver; **(C)** PAR1 protein expression in mouse liver; **(D)** CCL2 protein expression in mouse liver; **(E)** Expression of IP-10 protein in mouse liver. *p* values were determined using one-way ANOVA or an unpaired t-test. Data are expressed as mean ± SEM (n = 6). *p < 0.05, **p < 0.01, ***p < 0.001, ****p < 0.0001, ns means no statistical difference.

When TF was blocked, even with high expression of YKL-40, the mRNA levels of TF, PAR1, CCL2, and IP-10 did not show significant differences compared to untreated normal mice. However, the mRNA levels of TF, PAR1, CCL2, and IP-10 in the drug-induced liver injury group mice induced by ConA were significantly higher than those in the other three groups (Figure 6). The results of protein expression analysis are consistent with those of mRNA expression analysis (Figure 7). In summary, these findings suggest that blocking the TF-PAR1 pathway inhibits the release of downstream chemokines CCL2 and IP-10 mediated by YKL-40, thereby affecting the process of DILI.

### 3.6 Blocking TF-PAR1 pathway alleviates DILI progression

**FIGURE 8:**
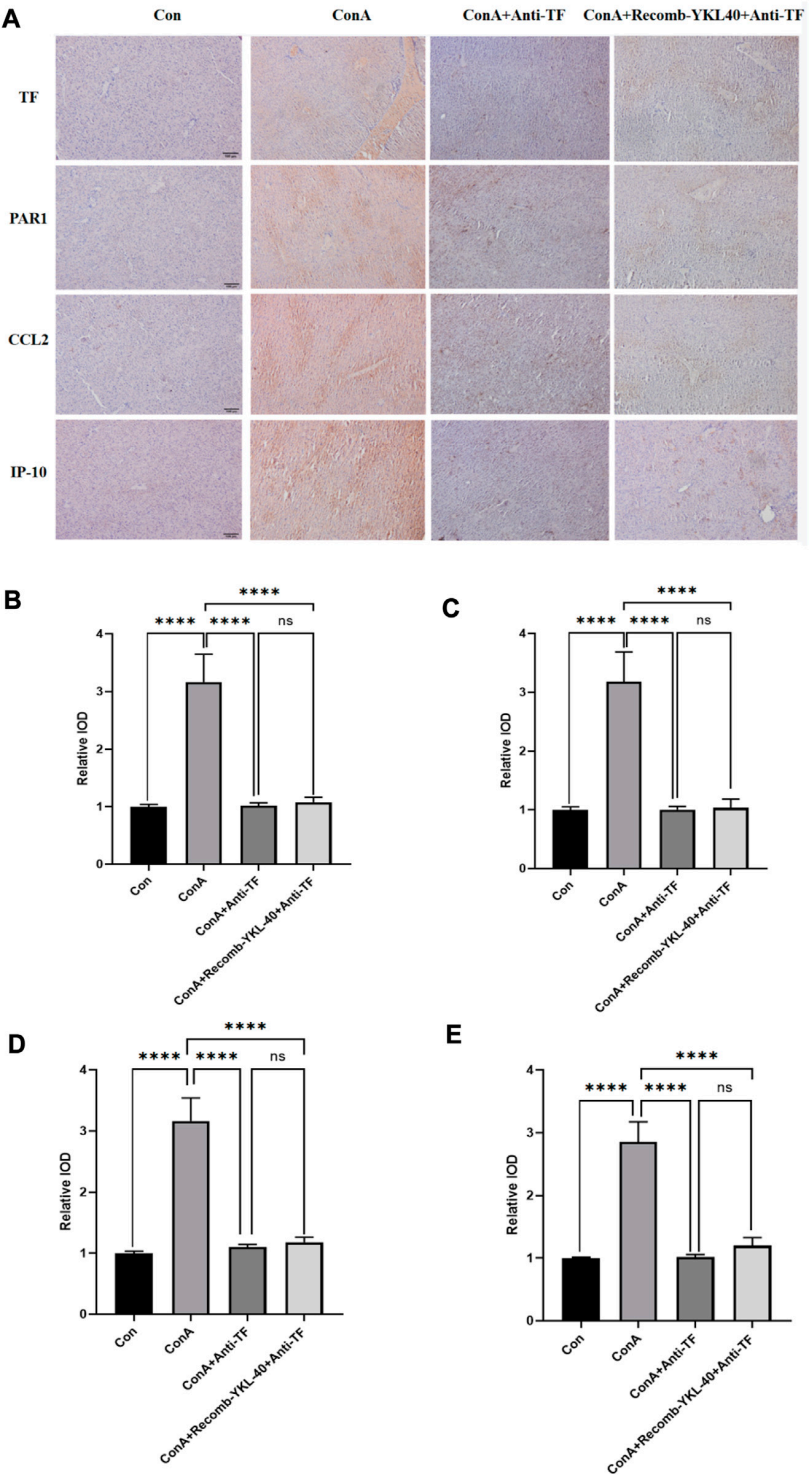
Blocking TF-PAR1 pathway alleviates DILI progression. **(A)** Blocking TF-PAR1 pathway alleviated drug-induced liver injury (DILI) as determined by immunohistochemistry (magnification×100) (n = 6). **(B)** Immunohistochemical statistics of TF; **(C)** Immunohistochemical statistics of PAR1; **(D)** Immunohistochemical statistics of CCL2; **(E)** Immunohistochemical statistics of IP-10. *p* values were determined using one-way ANOVA or an unpaired *t*-test. Data are expressed as mean ± SEM (n = 6). ^*^ *p* < 0.05, ^**^ *p* < 0.01, ^***^ *p* < 0.001, ^****^ *p* < 0.0001, ns means no statistical difference.

Inflammation and coagulopathy have been linked in several systemic inflammatory diseases such as sepsis and acute respiratory infections. Interleukin-1 (IL-1), IL-6, C-reactive protein and other inflammatory mediators stimulate TF expression on the surface of endothelial cells under inflammatory conditions such as sepsis and acute lung injury, leading to hypercoagulability (Abraham, 2000; Lou et al., 2019). At the same time, TF acts on endothelial cells via the PAR1 pathway to produce pro-inflammatory effects (Chen et al., 2004; Lou et al., 2019). Thus, highly expressed TF can play a dual role in promoting inflammation and coagulation. To verify whether inhibition of the TF-PAR1 pathway could inhibit the YKL-40-induced inflammatory immune response and attenuate the extent of liver injury in DILI by blocking the expression of downstream chemokines CCL2 and IP-10, we injected anti-TF antibodies in the model group and the corresponding YKL-40 high expression group blocked the expression of endogenous TF protein, and further histological examination was performed. Compared to the control group, blocking TF expression did not result in significantly increased expression of TF, PAR1, CCL2, and IP-10 even when YKL-40 was overexpressed; the model group (ConA group) had higher expression levels of all four markers in the liver than the other two groups that blocked TF and the control group (Figure 8). In conclusion, these findings suggest that blocking TF expression suppresses YKL-40-mediated expression of CCL2 and IP-10 through inhibition of the TF-PAR1 pathway.

## 4 Discussion

Drug-induced liver injury (DILI) is a liver injury caused by the drug itself and its metabolites during drug use. Drug specific heterogeneous reactions are currently a hot research topic. Drug-induced liver injury through direct toxic effects is often predictable, and its diagnosis of liver injury is relatively easy. However, drugs can cause liver damage through specific heterogeneous reactions, both in terms of mechanism and symptoms, which are particularly complex. Among them, allergic specific DILI works together with the body’s immune system, also known as immune mediated DILI. Due to its independence from dosage, unpredictability, and uncertain incubation period, diagnosis is even more difficult. The pathological process of the liver injury model established by Concanavalin A (ConA) is similar to that of many known acute and chronic liver diseases, especially it can better simulate the pathogenesis of human autoimmune liver diseases. Intrahepatic vascular coagulation plays an important role in the occurrence and development of liver diseases such as fibrosis, cirrhosis, and liver cancer. Therefore, in the context of relevant research, further exploration of the pathogenesis of DILI from the perspective of coagulation can contribute to a deeper understanding of immune mediated DILI.

The study revealed the relationship between inflammatory reaction and blood coagulation in systemic inflammatory diseases (including Sinusitis, acne vulgaris and acute hepatitis) (Montgomery et al., 2017; Shan et al., 2018; Ebrahim et al., 2020). More and more evidence suggests that after the coagulation system is activated in liver disease, some coagulation related proteins act as liver inflammatory factors. IAOC plays a crucial role in acute liver injury (Ganey et al., 2007; Sullivan et al., 2013; Miyakawa et al., 2015). In the ConA induced acute liver injury model, YKL-40 induced TF to participate in IAOC; It is worth noting that this process may be related to the activation of the MAPK pathway (Shan et al., 2018). Although the relationship between YKL-40 and TF has been elucidated, the mechanism by which YKL-40 regulates the downstream deterioration of liver diseases caused by TF is still unknown. Clarifying the interaction between the coagulation system and liver inflammation will provide new therapeutic targets for DILI. Therefore, in this study, we investigated YKL-40 mediated TF induction and its impact on the liver.

TF expression is often upregulated in inflammatory liver disease (Hisada et al., 2016; Henderson et al., 2021). The role of TF in initiating coagulation and thrombosis has been fully demonstrated. TF promotes coagulation cascade reactions by binding and activating coagulation factor VIIa (FVIIa) (Toschi et al., 1997). Secondly, in inflammatory diseases, TF exposure to the blood affects cell proliferation, apoptosis, scorching, migration, and aggregation of inflammatory factors through the TF-PAR1 pathway, ultimately exacerbating the vicious cycle of the “coagulation inflammation network” (Witkowski et al., 2016), thereby exacerbating the disease. PAR1, as the main thrombin receptor, is closely related to TF expression and plays a key role in liver fibrosis (Gutiérrez-Rodríguez and Herranz, 2015; Flaumenhaft and De Ceunynck, 2017). In recent years, it has been considered a new therapeutic target for inflammatory diseases. For example, after injecting PAR1 activating peptide into mice, thrombin can produce pro-inflammatory effects, manifested as edema and increased vascular permeability (Cirino et al., 1996). The formation of TF-FVIIa complex produces thrombin, which can activate cells through PAR1, thereby exacerbating the disease (Ganey et al., 2007). For example, PAR1 deficient mice have a significant response to Paracetamol (APAP) induced acute Liver failure after 6 h, which is manifested by liver injury and decreased liver fibrin deposition. Similarly, our findings suggest that YKL-40 activates the TF-PAR1 pathway and plays a crucial role in the progression of DILI. In addition, overexpression of YKL-40 is positively correlated with the expression levels of TF and PAR1, which can lead to intrahepatic coagulation and exacerbation of the condition. On the contrary, blocking the endogenous TF pathway will significantly reduce the mRNA and protein levels of TF and PAR1, as well as alleviate liver injury, even when YKL-40 is overexpressed. Similarly, we know that the increased expression of the inflammatory factor YKL-40 is significantly correlated with the severity of inflammatory diseases (Arain et al., 2020; Ebrahim et al., 2020). Our research results indicate that YKL-40 in the liver can aggravate liver injury and affect the progression of DILI by activating the TF-PAR1 pathway.

Chemokines are important inflammatory mediators that mobilize, guide, and locate effector cells in immune system mediated liver diseases. They also play a crucial role in controlling viral infections (Czaja, 2014). In addition, overexpression of chemokines can exacerbate the inflammatory response and increase the degree of liver damage. If its expression is effectively controlled, it will help regulate immune response and inflammation, thereby improving clinical symptoms and prognosis. In recent years, research has shown that chemokines CCL2 and IP-10 are closely related to liver diseases such as hepatitis, fibrosis, and cancer, which has attracted widespread attention. Among them, CCL2, as one of the key chemokines involved in liver inflammation and fibrosis, plays a crucial role in liver diseases (Mascia et al., 2017; Tacke, 2017). It has been widely validated in the hepatitis experimental model of CCL2^−/−^ mice (Karlmark et al., 2009; Galastri et al., 2012). CCL2 accumulation in murine model of liver fibrosis significantly increases the local inflammatory cell pool, inducing chronic inflammation that leads to further liver cell damage and stress responses, such as extracellular matrix deposition, steatosis, enhanced angiogenesis and the formation of an inflammatory microenvironment (Karlmark et al., 2009; Baeck et al., 2012; Baeck et al., 2014; Shao et al., 2022). IP-10/CXCL10 is a newly discovered chemokine that can combine with chemokine receptors expressed on NK cells and T cells to induce their migration to specific tissues. Its serum level is intimately related to the extent of necrotizing liver inflammation and fibrosis (Tacke et al., 2011; Elemam et al., 2022). For example, Hepatitis C (HCV) virus infection and intrahepatic INF-γ drive increased CXCL10 expression through the sinus endothelium and liver cells.

This induces T cell recruitment into the liver and aggravates the degree of necrotizing inflammation and fibrosis (Ferrari et al., 2019). IP-10 is also involved in immune system-mediated liver injury as observed in severe hepatitis. Its abnormally high expression drives the infiltration of several inflammatory cells into the liver tissue and initiates liver tissue necrosis (Karin and Razon, 2018). For instance, in humans, acute hepatitis B virus infection is accompanied by systemic cytokine and chemokine responses combined with increased serum CXCL10 level, which attracts more inflammatory cells to the liver and aggravates liver injury (Moroz et al., 2019). The interaction of the core protein and globular C1q receptor in HCV can induce macrophages to secrete CCL2 and CXCL10 through the NF-κB signaling pathway, thereby influencing the course of HCV (Song et al., 2021). CCL2 and IP-10 activation is reportedly regulated by the PAR1 pathway. In our study, YKL-40 overexpression following drug-induced injury activated the TF-PAR1 pathway, which in turn increased the expression of downstream chemokines CCL2 and IP-10 and aggravated liver injury. Suppressing TF-PAR1 pathway via blocking endogenous TF alleviates liver pathogenesis, even if YKL-40 is overexpressed. Therefore, TF-PAR1 pathway, which can be induced by YKL-40, plays a critical role in DILI. An in-depth investigation of the relationship between these pathways and chemokines can help us understand how leukocytes enter the liver and aggravate the immune response. This can also facilitate identification of therapeutic targets for liver inflammation. The various pathogenic mechanisms underlying liver diseases are not mutually exclusive, and in fact, may even be synergistic. Thus, further research is needed to determine the interaction among these pathways, their influence, and if they play unique roles at different stages of DILI.

## 5 Conclusion

This study revealed that YKL-40 promotes the expression of CCL2 and IP-10 in ConA-induced DILI in wild-type mice by inducing the TF-PAR1 pathway. This causes increased inflammatory cell recruitment, thus sparking the onset and progression of liver injury. This is a potential mechanism underlying the aggravation of liver inflammation and injury and presents novel targets for therapeutic intervention in DILI.

## Data Availability

The original contributions presented in the study are included in the article/Supplementary Material, further inquiries can be directed to the corresponding author.

## Abbreviations

TF, Tissue factor; IAOC, intrahepatic vascular coagulation; ConA, concanavalin A; PAR1, protein kinase 1; Chi3l1, inflammatory factor YKL-40; DILI, drug-induced liver injury; CCL2, Chemokine ligand 2; HCV, Hepatitis C.
