# Corrigendum: YKL-40 promotes chemokine expression following drug-induced liver injury via TF-PAR1 pathway in mice

**DOI:** 10.3389/fphar.2024.1395496

**Published:** 2024-08-15

**Authors:** Zhan Jing-Lun, Chai Shuang, Zhao Li-Mei, Liu Xiao-Dong

**Affiliations:** ^1^ Department of Pharmacy, Shengjing Hospital of China Medical University, Shenyang, China; ^2^ Department of the Second Clinical Pharmacy, School of Pharmacy, China Medical University, Shenyang, China

**Keywords:** YKL-40, TF-PAR1 pathway, inflammation, liver injury, CCL2, IP-10

In the published article, there was an error Figure 3A and its legend as published. The TF was inadvertently misused during the final assembly of Figure 3A. The corrected Figure 3 its caption appears below.

**FIGURE 3:**
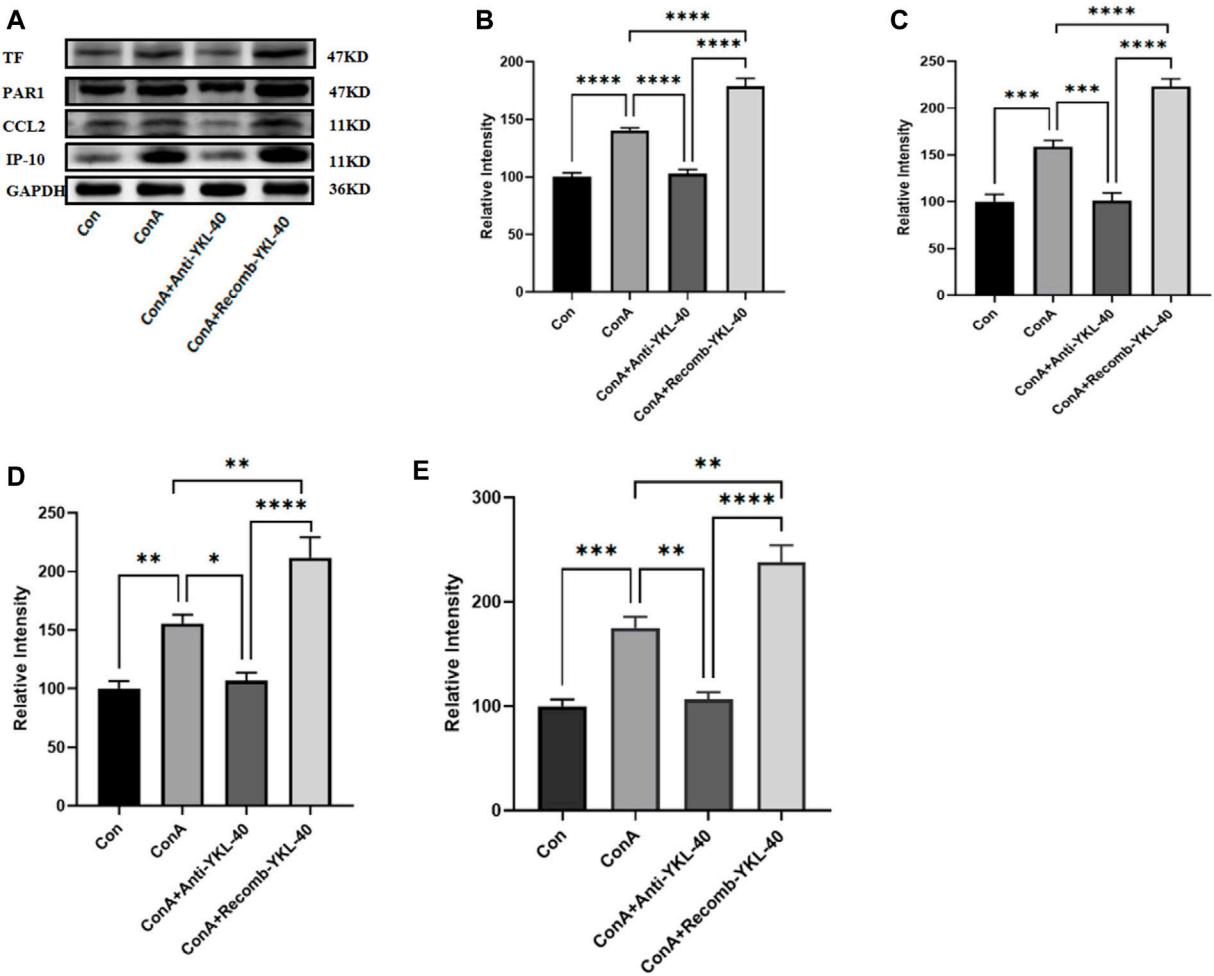
YKL-40 induces TF-PAR1 pathway and affects liver pathogenesis. **(A)** The levels of TF-PAR1 pathway proteins induced by YKL-40 in the liver and CCL2 and IP-10 were measured; **(B)** TF protein expression in mouse liver; **(C)** PAR1 protein expression in mouse liver; **(D)** CCL2 protein expression in mouse liver; **(E)** IP-10 protein expression in mouse liver. *p* values were determined using one-way ANOVA or an unpaired t-test. Data are expressed as mean ± SEM (n = 6). *p < 0.05, **p < 0.01, ***p < 0.001, ****p < 0.0001, ns means no statistical difference.

In the published article, there was an error Figure 7A and its legend as published. The TF was inadvertently misused during the final assembly of Figure 7A. The corrected Figure 7 its caption appears below.

**FIGURE 7:**
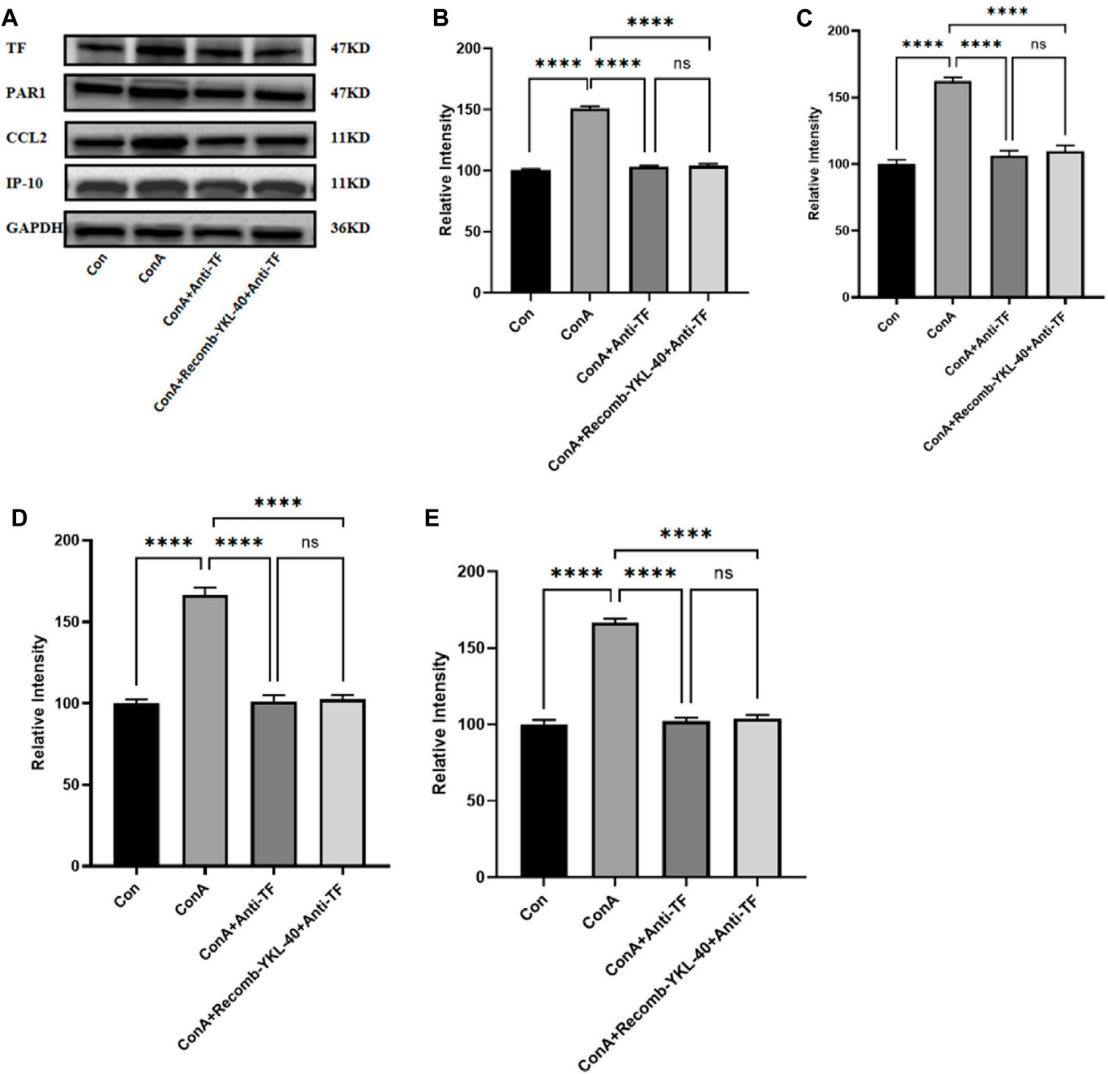
Effect of blocking TF-PAR1 pathway on liver pathogenesis. After the endogenous TF protein expression was blocked, **(A)** the expression of TF-PAR1 pathway proteins in the liver following YKL-40 induction and the levels of downstream chemokines CCL2 and IP-10 were determined; **(B)** TF protein expression in mouse liver; **(C)** PAR1 protein expression in mouse liver; **(D)** CCL2 protein expression in mouse liver; **(E)** Expression of IP-10 protein in mouse liver. *p* values were determined using one-way ANOVA or an unpaired t-test. Data are expressed as mean ± SEM (n = 6). *p < 0.05, **p < 0.01, ***p < 0.001, ****p < 0.0001, ns means no statistical difference.

In the published article, there was an error. The anti-TF antibody’s manufacturer and batch number are incorrect.

A correction has been made to **2. Materials and methods**, *2.1 Animal experiments*, Paragraph Number 1. This sentence previously stated:

“In the experimental groups, mice were immediately injected with recombinant Chi3l1 (500 ng, Sino Biological, 50929-M08H), anti-Chi3l1 antibody (500 ng, Sino Biological, 50929-RP01), or anti-TF antibody (1/1,000, 500 μg, ProteinTech, 17435-1-AP) after receiving ConA (**Shan et al., 2018**).”

The corrected sentence appears below:

“In the experimental groups, mice were immediately injected with recombinant Chi3l1 (500 ng, Sino Biological, 50929-M08H), anti-Chi3l1 antibody (500 ng, Sino Biological, 50929-RP01), or anti-TF antibody (1/1,000, 500 μg, Bioss, bs-4690R) after receiving ConA (**Shan et al., 2018**).”

In the published article, there was an error. The anti-TF antibody’s manufacturer and batch number are incorrect.

A correction has been made to **2. Materials and methods**, *2.4 Western blot analysis*, Paragraph Number 1. This sentence previously stated:

“The membrane was then blocked with a rapid blocking solution and incubated with the specific primary antibodies against TF (ProteinTech, 17435-1-AP, 1/3,000), PAR1 (Solarbio, K009690P, 1/1,500), CCL2/MCP-1 (ProteinTech, 25542-1-AP, 1/2,000), and CXCL10/IP-10 (ProteinTech, 10937-1-AP, 1/500).”

The corrected sentence appears below:

“The membrane was then blocked with a rapid blocking solution and incubated with the specific primary antibodies against TF (Bioss, bs-4690R, 1/1,000), PAR1 (Solarbio, K009690P, 1/1,500), CCL2/MCP-1 (ProteinTech, 25542-1-AP, 1/2,000), and CXCL10/IP-10 (ProteinTech, 10937-1-AP, 1/500).”

The authors apologize for these errors and state that this does not change the scientific conclusions of the article in any way. The original article has been updated.

